# The Fecal Virome of Children with Hand, Foot, and Mouth Disease that Tested PCR Negative for Pathogenic Enteroviruses

**DOI:** 10.1371/journal.pone.0135573

**Published:** 2015-08-19

**Authors:** Piyada Linsuwanon, Yong Poovorawan, Linlin Li, Xutao Deng, Sompong Vongpunsawad, Eric Delwart

**Affiliations:** 1 Center of Excellence in Clinical Virology, Chulalongkorn University, Bangkok, Thailand; 2 Blood Systems Research Institute, San Francisco, California, United States of America; 3 Department of Laboratory Medicine, University of California San Francisco, San Francisco, California, United States of America; University of California, San Francisco, UNITED STATES

## Abstract

Hand, foot, and mouth disease (HFMD) affects infant and young children. A viral metagenomic approach was used to identify the eukaryotic viruses in fecal samples from 29 Thai children with clinical diagnosis of HFMD collected during the 2012 outbreak. These children had previously tested negative by PCR for enterovirus 71 and coxsackievirus A16 and A6. Deep sequencing revealed nine virus families: *Picornaviridae*, *Astroviridae*, *Parvoviridae*, *Caliciviridae*, *Paramyxoviridae*, *Adenoviridae*, *Reoviridae*, *Picobirnaviridae*, and *Polyomaviridae*. The highest number of viral sequences belonged to human rhinovirus C, astrovirus-MLB2, and coxsackievirus A21. Our study provides an overview of virus community and highlights a broad diversity of viruses found in feces from children with HFMD.

## Introduction

Hand, foot, and mouth disease (HFMD) is an infectious disease that usually affects infants and young children under 5 years of age worldwide. HFMD typically causes self-limiting illness, but development of severe cardiopulmonary and neurologic complications have also been reported [1, 2]. The clinical manifestations are typically ulcerations in the oral cavity, buccal mucosa (enanthema) and tongue with peripherally distributed cutaneous lesions and vesicular rash (exanthema) on the palms of hands and soles of feet. Other parts of the limbs including knees, elbows and buttocks may also be affected. Transmission occurs via person-to-person through direct contact with respiratory secretion, saliva, fluid from blisters, and feces from infected individuals. A number of enteroviruses belonging to the family *Picornaviridae* cause HFMD, although human enterovirus 71 (EV71) and coxsackievirus (CV) type A16 are two of the most important enteroviruses implicated in many large-scale outbreaks in Asian-Pacific countries including Japan, Taiwan, Malaysia, Singapore, and China [2–4]. Additional enterovirus species including CV-A6, CV-A10 and CV-A4 also cause HFMD [5–9]. Clinical symptoms resulting from CV-A16 as well as other enteroviruses are usually relatively mild and indistinguishable with low incidence of severe complications. In contrast, serious complications such as encephalitis, myocarditis, and poliomyelitis-like illness were observed when EV71 were reported as the causative pathogen [10–12].

HFMD has been continuously present and remains a major cause of morbidity and mortality of young children particularly in Asia. Typically, HFMD exhibits cyclical pattern of outbreaks every two to three years. Factors underlying the prevalence of HFMD remain controversial. Data from countries with long history of HFMD outbreaks suggest that dissemination is associated with socio-economic status, population ethnicity [13], regional climate [14–16], and attendance in school or care centers of school-age children [17]. The magnitude of HFMD outbreaks appears to fluctuate [3, 18, 19]. HFMD in tropical climate countries, such as Malaysia, Singapore and Thailand, typically showed years-round activity with no discrete epidemic periods although peaks during the rainy and winter seasons were also detected depending on season, year, and geographic regions [20, 21]. At present, no specific treatment for HFMD exists. Vaccines or antiviral drug against EV71 are currently being developed in Taiwan, China and Singapore but are not yet commercially available [22–24]. Prompted by geographically widespread outbreaks, careful monitoring of the spatial and temporal epidemiology is considered to be of great importance to control the spread of HFMD according to the different regional characteristics. As reported by the Bureau of Epidemiology, Ministry of Public Health of Thailand, HFMD has shown an upward trend in the last five to six years. In June 2012, the largest recorded outbreak of HFMD occurred throughout the country. This outbreak affected more than 39,000 individuals, including three deaths, over a period of four to five months with hot spots in Chiang Rai and Mae Hong Son provinces [25]. In our previous study, we monitored HFMD activity in Thailand between 2008 and 2012. Our results revealed that the HFMD epidemic in 2012 was significantly different from previous ones in Thailand including the size of the epidemic and the viruses detected [26, 27]. During the 2012 epidemic, beside a high prevalence of EV71 and CV-A16, multiple EV types such as CV-A6 were also detected. Even though the standardized 5' untranslated region (5'UTR) pan-enterovirus PCR and viral capsid protein 1 (VP1) gene typing PCR assays were used [27], approximately one third of the suspected cases, mostly young children, were negative for enterovirus by these assays. These findings raised questions regarding the sensitivity of the current assay in identifying causative viruses other than EV71 and CV-A16 that may be present below the limit of detection by conventional PCR. In recent years, metagenomic has become an important strategy for virus discovery in human and animal diseases [28–30]. This technique, based on recognition of sequence similarities following non-specific nucleic acid amplification, circumvents some of the limitations of virus isolation, serology, and the amplification of only known conserved genomic regions [31]. To evaluate circulating enterovirus and previously uncharacterized viruses associated with HFMD, we describe here the virus community (virome) in fecal samples negative by RT-PCR for EV71 and CV-A16/A6 obtained from 29 pediatric patients with HFMD during the outbreak in Thailand in 2012.

## Materials and Methods

### Sample preparation for metagenomic analysis

The research protocol was approved by the Institutional Review Board of the Faculty of Medicine, Chulalongkorn University. Since the samples were anonymized and could not be traced back to individual patients, the ethical board determined that the need for informed consent was waived. All clinical investigation was conducted according to the principles expressed in the Declaration of Helsinki.

A total of 29 fecal samples were selected from a collection of archived samples obtained from children with HFMD who were admitted to medical centers and hospitals in Bangkok and Khon Kaen province during the nationwide outbreak in 2012. The inclusion criterion for patient enrollment has been described elsewhere [26, 32]. All samples were previously tested using a 5' UTR pan-enterovirus and EV71, CV-A16/A6 specific VP1 PCRs and found to be PCR negative for enteroviruses [27]. Demographic information of the patients for the 29 selected patients is summarized in Table 1. The HFMD patients were between 8 months and 5 years old (1 male: 1.4 female). Stool was suspended in 1x phosphate buffer saline (PBS) solution and subjected to a vigorous vortex to yield fecal supernatants. Then 140 μl of fecal supernatant from each of 29 HFMD patient samples were mixed into three pools, designated as HFMD-lib01 (10 samples), HFMD-lib02 (10 samples), and HFMD-lib03 (9 samples). The sample pools were centrifuged at 12,000x g and the supernatants were adjusted to 2 ml final volume using PBS. The pooled supernatants were filtered through a 0.45-μm membrane (Millipore, MA, USA) to remove eukaryotic- and bacterial-cell-sized particle and to enrich for viral contents. The filtrates were concentrated using ultracentrifugation at 22,000 x g for 2 hours and the pellets were resuspended with 100 μl 1X PBS. To eliminate host genome and other non-viral nucleic acids, a mixture of DNases [Turbo DNase (Life Technologies, NY, USA) and Baseline-ZERO (Epicentre, WI, USA)] and RNase (Fermentas, Ontario, Canada) were added to 120 μl of the concentrated suspension to the final volume of 140 μl followed by enzymatic digestion at 37°C for 1.5 hours. Viral particle-protected nucleic acids were then extracted using the QIAamp viral RNA extraction kit (Qiagen, Hilden, Germany) according to manufacturer’s instruction. Total nucleic acids were eluted in 25μl RNase free water (Life Technologies, NY, USA).

**Table 1:** Summary of sequencing data of eukaryotic viruses and picornaviruses detected in HFMD pool samples.

	Eukaryotic viruses detected		Picornaviruses detected	
Library Sample	List of Virus	Read	List of Virus	Read
HFMD-lib01	Picornaviruses	1,244	Saffold virus	788
	Bocaparvovirus	1,291	Coxsackie virus A10	433
	Astrovirus	10	Coxsackie virus A16	17
	Calicivirus	7	Enterovirus 71	3
	Adenovirus	9	Enterovirus 68	3
	Reovirus	30		
HFMD-lib02	Picornaviruses	1,784	Rhinovirus C	1,772
	Calicivirus	11	Coxsackie virus A8	6
	Adenovirus	3	Saffold virus	4
			Coxsackie virus A21	2
HFMD-lib03	Picornaviruses	1,678	Coxsackie virus A21	1,334
	Astrovirus	1,469	Coxsackie virus B1	125
	Calicivirus	703	Enterovirus B	200
	Paramyxovirus	187	Echovirus 3	17
	Adenovirus	21	Enterovirus A	2
	Picobirnavirus	29		
	Polyomavirus	6		

### Library construction for deep sequencing

DNA for sequencing were prepared by using a ScriptSeq RNA-Seq library preparation kit (Epicentre, WI, USA) according the manufacturer’s recommendation. Terminal-tagged oligomer were subsequently annealed at the 3′-end. The di-tagged single strand cDNAs were purified using 8x volume of Agencourt AMPure magnetic-bead (Beckmann Coulter, CA, USA) and used as starting templates for second-strand synthesis. The libraries were enriched by 15 cycles of PCR amplification. During the amplification, different tagged random primers containing short nucleotides signature at the 3′-end of the primers (barcodes) were used to assign sequences to the corresponding pool. Finally, amplified adaptor-tagged libraries were further cleaned up by using Agencourt AMPure magnetic bead purification followed by library quantification using KAPA library quantification kit (Kapa Biosystems, MA, USA). The purified products from the three pools were combined in equal concentrations and subjected to sequencing on the MiSeq Illumina platform (Illumina, CA, USA)

### Data processing and analysis

Pair-end products of 250 bp in length were automatically binned into 3 groups according to their barcodes using in-house bioinformatics analysis pipeline [33]. The primers and low quality sequences were trimmed and the remaining sequences assembled into contigs using Sequencher program with the assembly criteria described previously [34]. The assembled contigs and singlet sequences longer than 100 bp were queried for sequence similarity using BLASTn, BLASTx and tBLASTx against non-redundant nucleotide and protein databases. Sequences were classified according to their closest BLAST match if they were found to have 80% sequence coverage with a stringent e-value ≤ 1E^-5^.

### Recombination analysis

To determine whether the virus sequences identified in this study contained any potential recombinants, nucleotide sequences of viruses were retrieved from GenBank and ViPR databases (November, 2014). The sequences were aligned by using MAFF program (v7.0) to adjust for the correct open reading frame [35]. Prediction of recombination event was performed using Recombination Detection Program (RDP v4.36) with the parameter setting as previously described [36]. Potential recombination event was considered if the subsequent event from two or more analyses were found with statistically significant support (P-values < 1E^-5^). In addition, crossover site was consistently checked by performing similarity plot and boot-scanning analysis using SimPlot software.

### Specific PCR for taxonomic species assignment of deep sequencing-derived contigs confirmation

To confirm the taxonomic species assigned to the contig sequences by deep sequencing, semi-nested PCR was done on the original fecal samples comprising the pools. Enteroviruses were detected using concensus-degenerate hybrid oligonucleotide primers specific towards the VP1 gene according to a previously described amplification protocol [27, 37]. The detection of rhinovirus was performed using the primer targeting 5′UTR through the VP2 gene as previous described [38].

### Phylogenetic analysis

To evaluate the genetic relationship among the virus-related sequences within the same species and/or family, phylogenetic trees were constructed using the neighbor-joining method with maximum composite likelihood nucleotide substitution model and pairwise deletion for missing data implemented in MEGA program (v5.2.2). Branch support and nodal confidence were assessed by bootstrap resampling with 1,000 replicates and bootstrap value of 70% was used as the cut-off for cluster analysis. Percent nucleotide and amino acid sequence identities from pairwise comparison were calculated using BioEdit Sequence Alignment Editor (v7.0.9.0).

## Results

**Fig 1:**
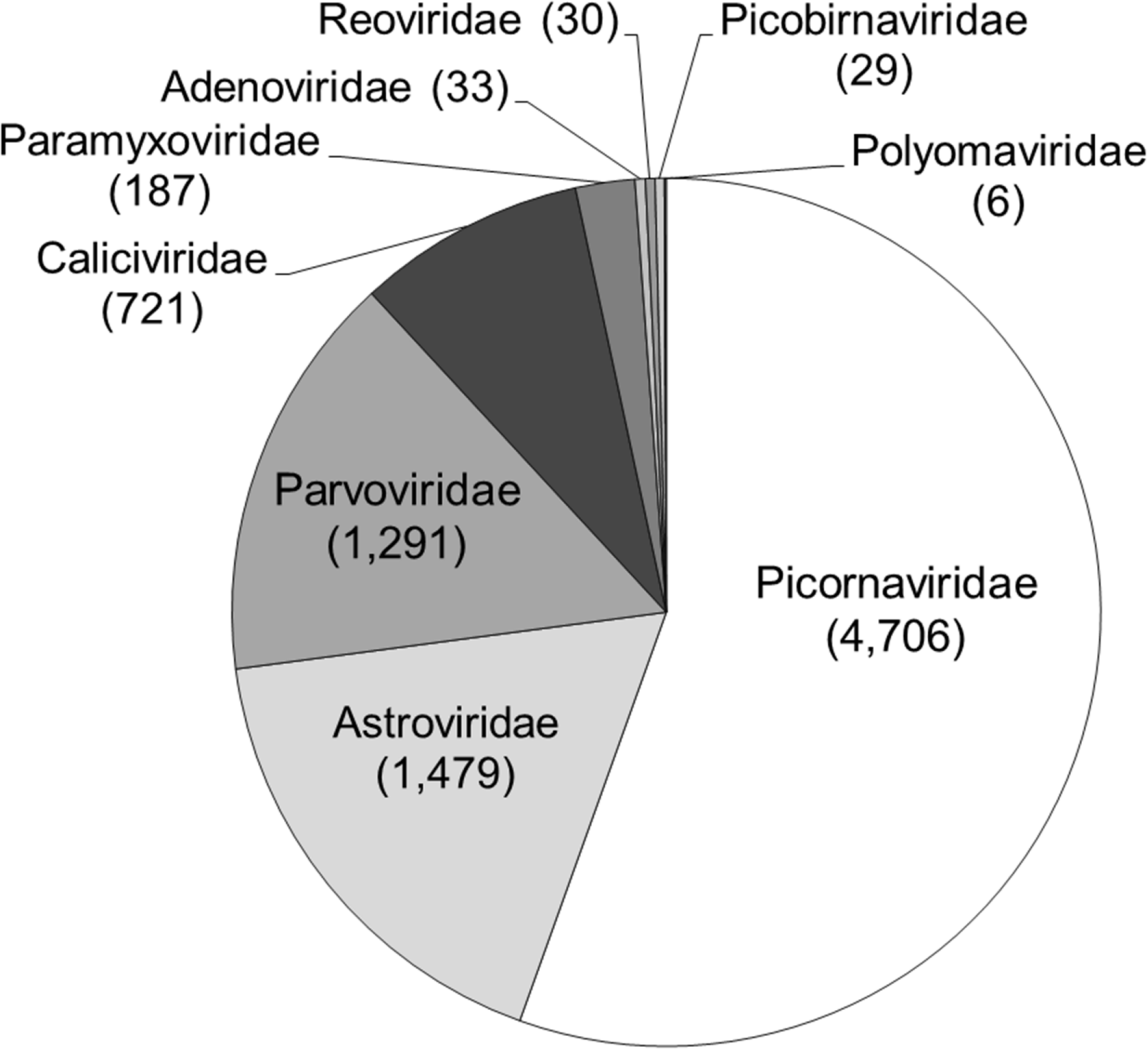
The distribution of the different viral families found in the fecal samples of patients with HFMD. The viral sequences identified from the pooled samples were grouped based on the closest homolog and arranged into families. The derived number of reads was noted in the parentheses.

Since the amplification of virus sequences was performed using pooled specimens, virus-related sequences identified in this study would be referred for virus population in the pools rather than per individual specimens. Contigs and reads related to viral origin were further sorted into families and species based on BLAST homologous classifications. The number of sequence reads was directly proportional to the relative abundance of DNA in the original samples, our study therefore compared the number of reads derived from the assembled contigs rather than the number of contigs alone. Virus enrichment followed by random nucleic acid amplification and MiSeq Illumina sequencing yielded 8,482 sequence contigs and singlets from HFMD patients that showed similarity to eukaryotic viruses, comprising 2,591 sequences from HFMD-lib01, 1,798 sequences from HFMD-lib02 and 4,093 sequences from HFMD-lib03. The sequences could be classified into nine viral families and twelve species including six families of RNA viruses: *Picornaviridae*, *Astroviridae*, *Caliciviridae*, *Paramyxiviridae*, *Reoviridae*, and *Picobirnaviridae*, and 3 families of DNA viruses: *Parvoviridae*, *Adenoviridae*, and *Polyomaviridae*. The proportions of the identified virus families in decreasing number of reads are summarized in Fig 1 and general information of the sample in the HFMD pool is shown in S1 Table.

### Viral families found in HFMD

#### Family *Picornaviridae*

The family *Picornaviridae* consists of 29 ICTV-approved genera, many of which are implicated in a wide range of infectious diseases in human and animals. Picornaviruses are non-enveloped, icosahedral viruses with single-stranded RNA segments (ssRNA) of positive polarity that typically range in size from 7 to 9 kb. In the present study, picornaviruses constituted the majority of the virome sequences, accounting for 55.5% (4,706/8,482) of the HFMD eukaryotic viral sequences (Fig 1). Most picornavirus sequences in the HFMD pools were from the genus *Enterovirus* [83.2% (3,914/4,706)], including enterovirus and rhinovirus species. The remaining sequences were human saffold virus from the genus *Cardiovirus* [16.8% (792/4,706)].

#### Human Enterovirus

**Fig 2:**
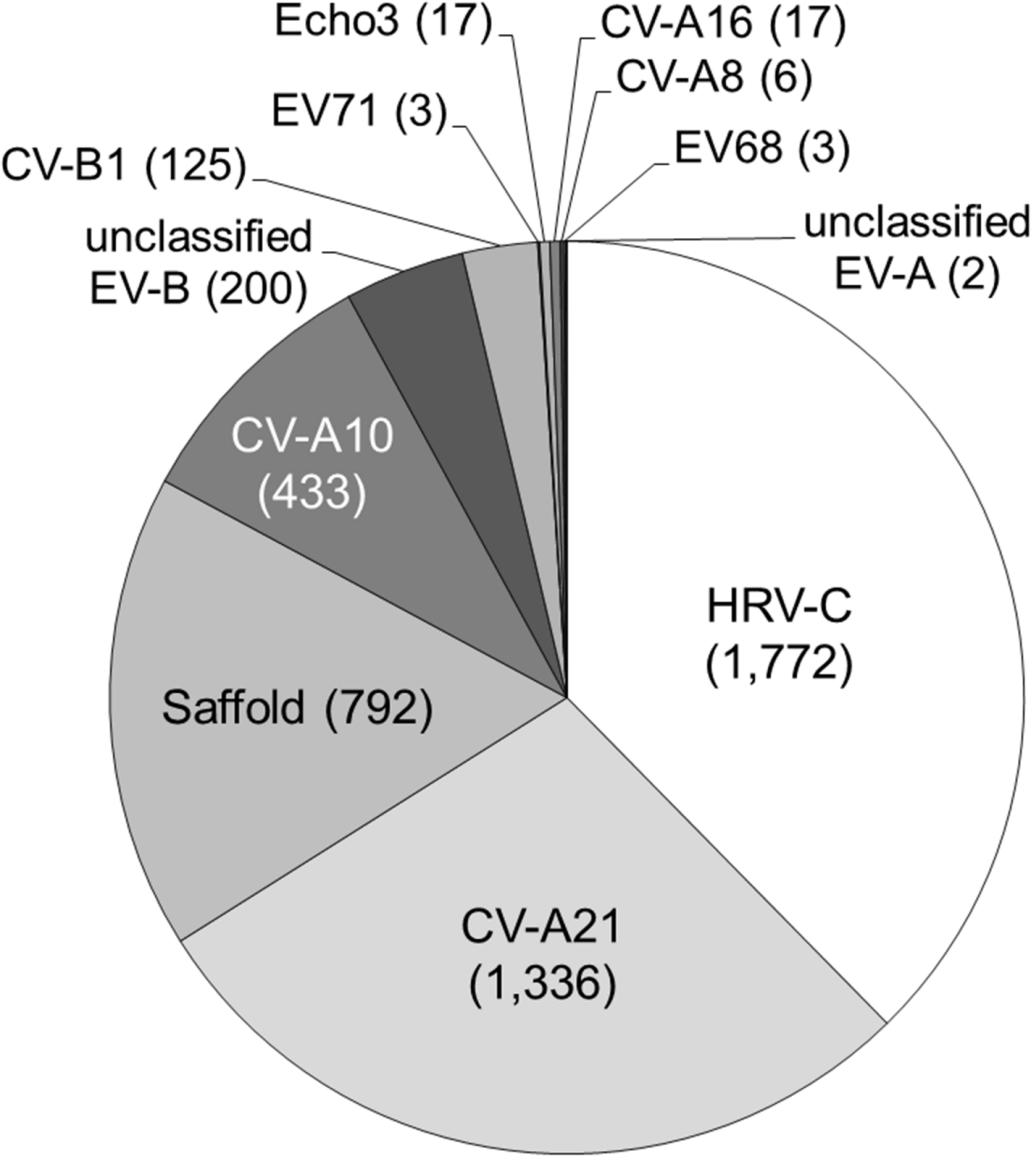
An overview of the picornaviruses found in the HFMD samples. The numbers of reads when the nucleotide sequence alignment showed e-value ≤ 1E^-5^ were indicated.

**Fig 3:**
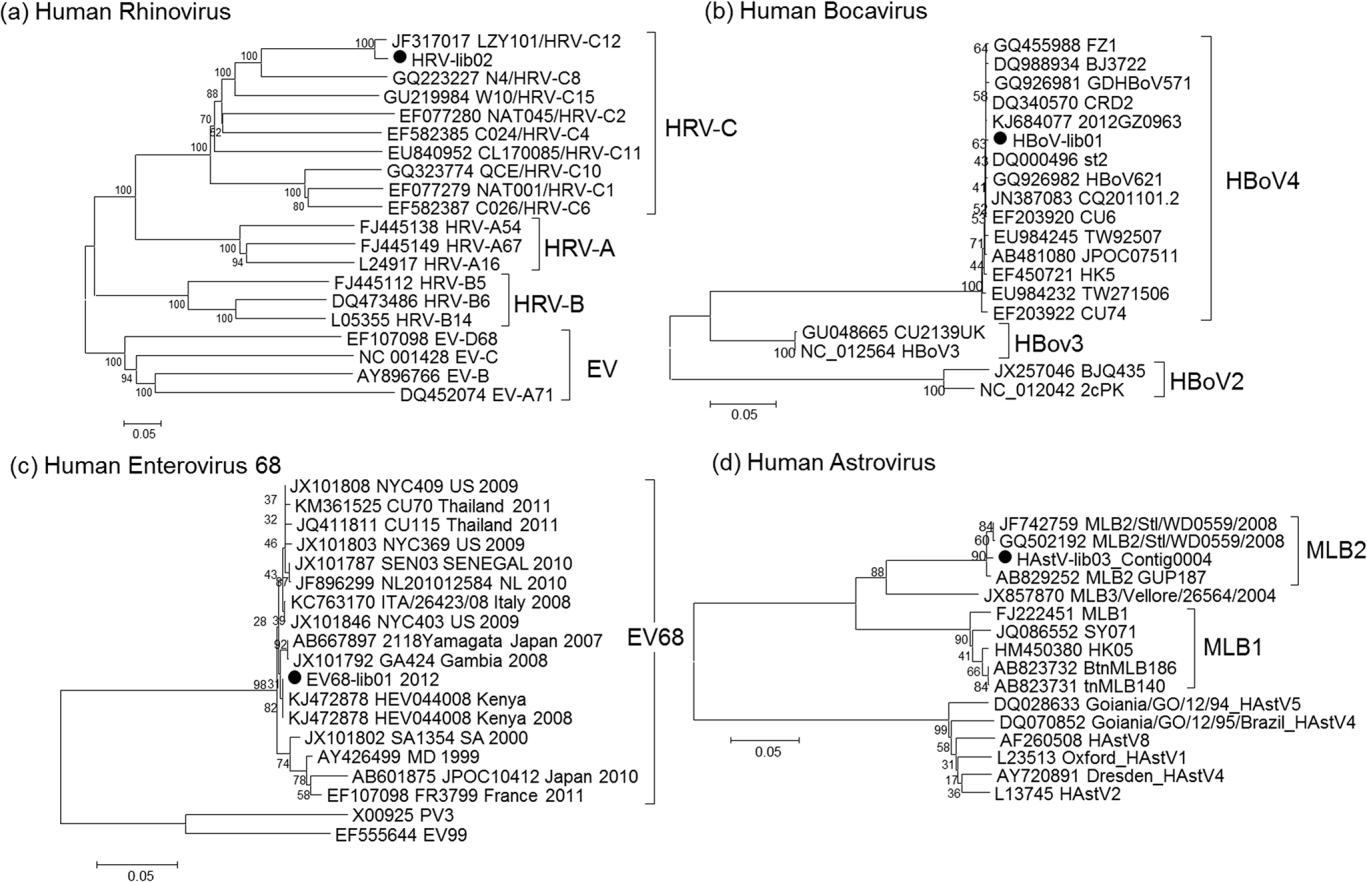
Neighbor-joining phylogenetic trees of the virus strains identified from the HFMD pools and their relationship to other related viruses. They include nearly complete genome sequence of (a) human rhinovirus strain “HRV-lib02” (6,892 nucleotides) and (b) human bocavirus strain “HBoV-lib01” (5,126 nucleotides), (c) partial capsid VP1 sequence of human enterovirus serotype 68 strain “EV68-lib01” (371 nucleotides) and (d) partial serene protease encoding sequence of human astrovirus strain “HAstV-lib03” (2,305 nucleotides). Sequences reported in this study were marked with black circles. Scale bar indicated the number of nucleotide substitutions per site.

The genus *Enterovirus* currently consists of nine enterovirus species (designated species A, B, C, D, E, F, G, H, and J) and 3 rhinovirus species. Only enterovirus (EV) species A to D and rhinovirus are human viruses. EV infections primarily cause diseases in the alimentary and respiratory tract, sometimes with manifestation in the central nervous system, most frequently in infants and young children [39]. EV-like sequences could be characterized abundantly in the pooled clinical samples including sequences most closely related to EV species A, B and D (2,142 reads) and rhinovirus (1,772 reads) (Fig 2). Approximately 45.9% (1,797/3,914) of enterovirus sequences were similar to several types within EV species A including CV-A21 [74.3% (1,336/1,797)] (Fig 2). In general, CV-A21 infection is thought to cause mild illness and is not typically associated with HFMD. The CV-A21 sequences identified in this study shared 80%-97% nucleotide identity, depending on the genomic region, to previous strains in the GenBank database. Other EV species A related sequences included 24.1% CV-A10 (433), 0.9% CV- A16 (17), 0.3% CV-A8 (6), and 0.2% EV71 (3) (Table 1). Sequences homologous to EV species B constituted 16% (342) of enterovirus reads, including CV-B1 (125) and echovirus (Echo) type 3 (17). On the basis of sequence similarity, another 200 reads were classified as EV-B but were unassignable to a specific type. In addition to EV BLAST analysis results, three nucleotide sequences from the lib01 pool, which could be assembled into a 371 nt long contig (accession number KP242036), were assigned to EV species D type 68 VP1 gene. Phylogenetic analysis showed that EV68-lib01clustered with the previous reported strain identified from many countries worldwide with more than 92% nucleotide identity (Fig 3).

#### Human Rhinovirus

Human rhinovirus (HRV) has a high level of genetic diversity as exemplified by the 80 types for HRV-A, 32 types for HRV-B, and at least 54 phylogenetically distinct lineages of HRV-C [40]. Infections by HRV are most often associated with respiratory tract infection from self-limiting to life-threatening illnesses [35, 41]. Furthermore, a number of recent studies demonstrated an association between HRV infections and gastroenteritis [42, 43]. In the present study, a total of 1,772 HRV-like sequences was recovered from HFMD-lib02 (HRV-lib02), accounting for 45.2% of the enterovirus reads. These sequences could be assembled into one long contig spanning 6,892 nucleotides (nt) representing 96% of the HRV genome (KP242035) spanning from a partial 5′UTR (581 nt) through the RNA-dependent RNA polymerase (RdRp) encoding gene (Table 2). HRV-related sequences displayed at least 96% nucleotide identity to the respiratory tract-derived HRV isolate LZY101 from China (JF317017) and could be phylogenetically classified as HRV-C12.

**Table 2:** Description of assembly results of the sequences of human rhinovirus, saffold virus, bocavirus, and astrovirus.

Sequence	Virus strain	Total Read	Genomic coverage	Coverage	Identity
HRV-lib02	HRV-C12	1,772	5'UTR to RdRp	96%	96%
SAFV-lib01	SAFV3	792	L protein, VP2 to 3C, 3C to 3'UTR	75%	98.6%
HAstV-lib03	MLB2	1,479	Serine protease to RdRp, Capsid proteins	93%	99%
HBoV-lib01	HBoV4	1,291	5′UTR to partial 3′UTR	99%	99%

Virus type or strain was assigned based on the BLAST analysis; Identity was selected from the highest nucleotide similarity to reference sequences; nt, nucleotide; RdRp, RNA-dependent RNA polymerase

Recombination is known to occur frequently among picornaviruses and is considered a major driving force of viral evolution and antigenic diversification [44]. Some HRV-C strains appear to originate from recombination between the ancestors of HRV-A and HRV-C in 5′UTR, resulting in the classification of two separate subspecies designated as HRV-Ca (sharing the 5′UTR with HRV-A) and HRV-Cc (possessing a unique 5′UTR) [45, 46]. Phylogenetic analyses of the viral capsid genes VP4/VP2 and VP1 and 5′UTR suggested that the HRV-related sequence strain from pooled HFMD clinical samples formed a unique branch with HRV-Cc subspecies. The recombination analysis of these HRV-related sequences showed no evidence of recombination. Characteristic features of HRV-C, including deletions in the BC and DE loops and nucleotide non-synonymous substitution in the immune-dominant VP1 gene that decreased viral susceptibility to the antiviral drug plecornaril (Y152 and T191 substitutions) [47, 48], were also identified in this strain.

#### Human Saffold Virus

Human saffold viruses (SAFV) are members of the genus *Cardiovirus*, family *Picornaviridae*. First identified in 2007 from a child with febrile illness, SAFV are found in the stool of children with nonpolio acute flaccid paralysis and healthy children in Pakistan [34]. However, pathogenicity and clinical relevance of SAFV infection remain to be fully elucidated. SAFV replicates efficiently in the alimentary tract and is transmitted through oral-fecal route, consistent with possible association with gastrointestinal tract illnesses and diarrhea [49]. Infections by SAFV might also contribute to more severe symptoms in children such as HFMD-associated encephalitis, acute flaccid paralysis, myocarditis, and aseptic meningitis [50–53]. In this study, SAFV-related sequences were found in two HFMD pools, contributing to the third most picornavirus sequence reads detected [16.8% (792/4,706)] after HRV-C and CV-A21. Amongst the SAFV-like sequences, two contigs (5,405 nt and 524 nt) and a singlet (176 nt) were recovered from a sample pool. Together, these sequences spanned approximately 75% of the entire genome and encompassed partial L protein encoding region (176 nt), VP2 through 3C (5,405 nt), and the 3′end of 3C through 3′UTR (524 nt) (Table 2). BLAST searches of full-length sequences and phylogenetic analysis of the individual regions suggest that these samples contained a genotype similar to the strain JPN404 from Japan (HQ902242). Sequences shared 87.1%-98.6% nucleotide identity with other SAFV3 cardioviruses. The overall nucleotide identity in the partial polyproteins 1 (P1) and P2 regions to other SAFV genotypes were 66.3%-68.9% and 72.2%-90.6%, respectively. Genome identity to SAFV3 were highest in VP2 (206 nt; 70%-99.5%)) and VP3 (696 nt; 71%-98%), and lowest with VP1 (813 nt; 62%-99.2%).

**Fig 4:**
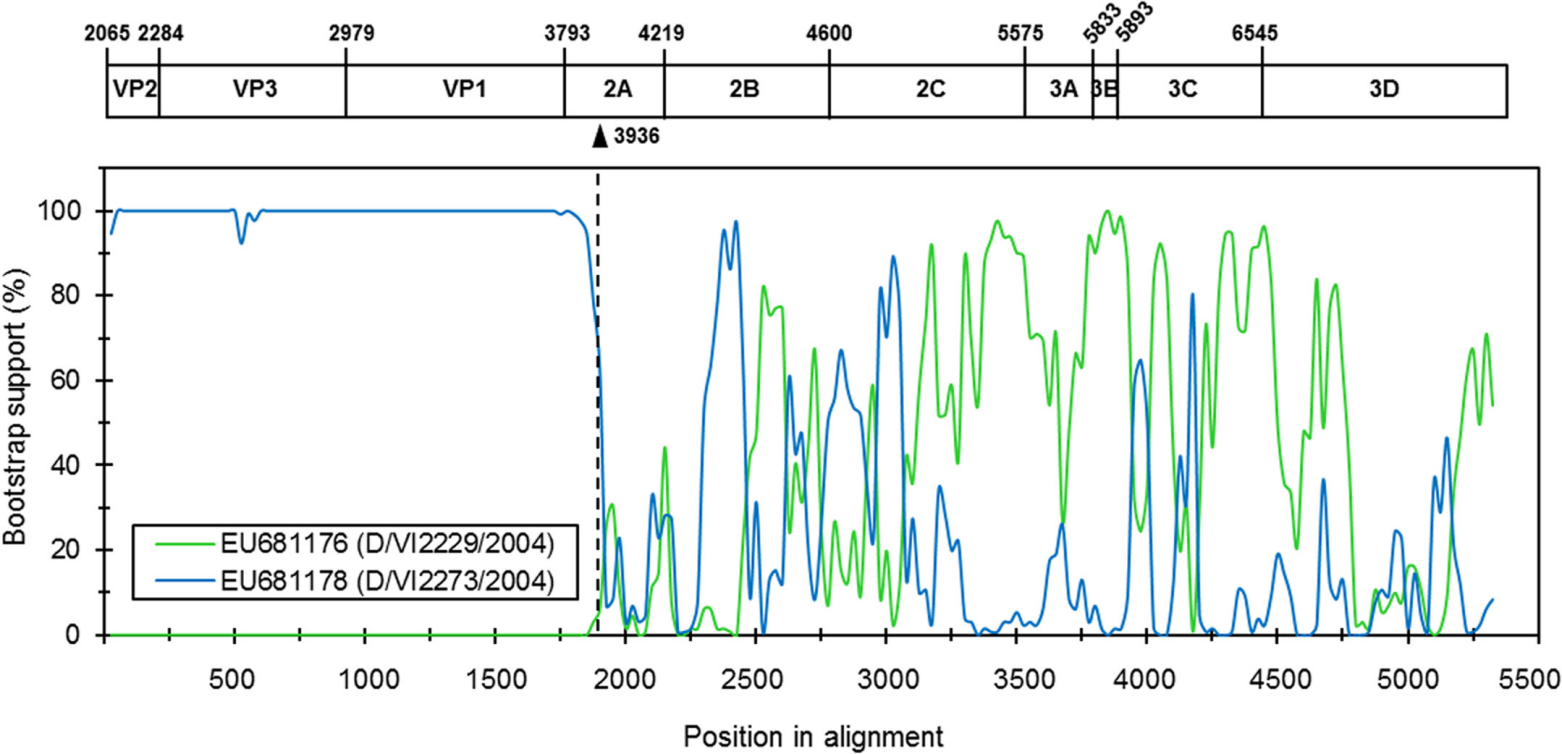
Potential intratypic recombination event in the nonstructural region of SAFV-like sequence. The x-axis indicated the nucleotide position of the alignment, and the y-axis showed the percentage of bootstrap pseudo-replicates that supported grouping of the query sequences with other SAFV. The potential recombination breakpoint near nucleotide 3,936 was labeled with a solid black triangle.

In order to investigate possible recombination event in the SAFV-like sequence identified from HFMD clinical sample pool, we performed a scan of the full reference set of completed and nearly completed genome sequences of eleven SAFV genotypes (41 sequences) and theilovirus-like members (13 sequences) using a suite of recombination detection program in RDP4. Results showed that the SAFV-like sequence appeared to result from intertypic recombination between SAFV2 (major parent: EU681176) and SAFV3 (minor parent: EU681178) (Fig 4, Table 3 and S2 Table). The SAFV-like sequence shared 93.8% nucleotide identity to SAFV3 in the P1 region (p < 0.05). These similarity patterns were also observed for the strain JPN404. Based on boot-scanning analysis, the presence of one putative recombination breakpoint was detected within the beginning of 2A gene (around nucleotide position 1,871 of the contig or position 3,936 of the strain JPN404). Our findings together with recent reports on SAFV genetic diversity [54, 55], suggest the important role of recombination in the emergence of SAFV genotypes and strains.

**Table 3:** Recombination analysis of SAFV using sequences from the reference set and the pooled samples from HFMD patients based on P-value.

Parental Strains									
Major	Minor	Recombinant	Breaking point	RDP	BootScan	MaxChi	Chimera	Siscan	3Seq
EU681176	EU681178	SAFV-lib01	VP2, 2A	2.12E-14	3.58E-45	5.89E-26	2.55E-24	5.54E-64	3.59E-24

P-value was calculated using Recombination Detection Program

#### Human Astrovirus

Human astrovirus (HAstV) is a small positive ssRNA virus which belonged to the *Mamastrovirus* genus, family *Astroviridae*. It is associated with gastroenteritis predominantly effecting young children [56]. We found astrovirus-related sequences in HFMD-lib01 and HFMD-lib03, constituting the second most abundant viral reads in the HFMD virome [17.4% (1,479/8,482)]. The relative ratio of HAstV-like sequences to the total number of virus reads was 35.9% in HFMD-lib03 (1,469/4,093) and 0.4% in HFMD-lib01 pool (10/2,591). HAstV-like sequence fragments identified from HFMD-lib01 pool covered approximately 18% of the genome. HAstV-lib03 (KP242038) provided nearly complete sequence spanning more than 93% of the genome including serine protease to RdRp (603 nt, 2,351 nt and 372 nt) and capsid protein encoding genes(2,493 nt) (Table 2). Phylogenetic analyses of the HAstV-like sequences demonstrated that the HAstV-lib01and HAstV-lib03 clustered within the new classified lineage of HAstV MLB1 and MLB2, respectively.

#### Human Bocavirus

Sequences relating to human bocavirus (HBoV) comprised the third most abundant viruses of the virome. HBoV belongs to the genus *Bocavirus* (subfamily *Parvovirinae*, family *Parvoviridae*) and can be classified into 4 genotypes designated as HBoV1 to HBoV4 by the structural VP1 sequence-based phylogenetic tree. HBoV plays a role in RTI and enteropathogenesis and may be associated with neurologic complications [57–59]. Currently, a new classification in the family *Parvoviridae* using phylogenetic relationship of the nonstructural gene DNA sequences has been proposed [60]. According to this proposed criteria, HBoV1 and HBoV3 have been re-categorized into the group of bocaparvovirus 1, while HBoV2 and HBoV4 are members of bocaparvovirus 2. HBoV-like sequences were found in the HFMD01 pool (HBoV-lib01), constituting 15.2% (1,291/8,482) of total virus reads in HFMD. The sequences could be assembled into single long contig covering 5′UTR to partial 3′UTR (5,271 nt) with 99% nucleotide sequence identity (KP242037) (Table 2). BLAST and phylogenetic analysis of the complete sequence length of HBoV-lib01 indicated that this virus was HBoV4 in bocaparvovirus 2 and shared more than 99% nucleotide identity to the previous reported strains.

#### Human Norovirus and Sapovirus

The *Caliciviridae* family of small, non-enveloped, positive ssRNA viruses now comprises five recognized genera: *Norovirus*, *Sapovirus*, *Lagovirus*, *Vesivirus*, and *Nebovirus*. Caliciviruses infect humans and animals of economic importance. Frequently, noroviruses (NoV) and sapoviruses (SaV) are responsible for outbreaks of nonbacterial gastroenteritis worldwide [61, 62]. Both NoV and SaV are each segregated into at least five genogroups (GI to GV) on the basis of sequence comparison of the RNA polymerase and capsid gene. The virome of the HFMD comprising viruses in the family *Caliciviridae* accounts for 8.5% (721/8,482) of the sequence reads consisting of 98.5% (710/721) and 1.5% (11/721) of SaV-related and NoV-related reads, respectively (Fig 1). Sequences relating to SaV were found in the pool HFMD-lib01 (7 reads) and HFMD-lib03 (703 reads). Nucleotide identity to the known strains of SaV varied between 94% to 99% for alignments covering between 114 nt to 1,032 nt. Despite the fewer number of sequence reads for NoV, the analysis of one contig (237 nt) and two singletons (129 nt and 165 nt) showed homology to NoV genogroup I. The NoV-like sequences shared at least 80% and 87% nucleotide identity to the known strains in ORF1 and ORF2, respectively.

#### Human Adenovirus

Human adenoviruses (HAdV), family *Adenoviridae*, are non-enveloped icosahedral viruses with a linear, double-stranded DNA genome. Infection by HAdV is associated with a wide spectrum of clinical diseases including RTI, gastroenteritis, and keratoconjunctivitis [63–65]. Seven HAdV species, designated A to G, are formally recognized. We found 0.4% (33/8,482) HAdV-related sequences in the HFMD sample pool. The amplified fragments were between 120 nt and 280 nt in length and covered several parts of the genome including the U exon, single-stranded DNA binding protein, DNA polymerase, E4 control protein, and E1B large T antigen encoding regions. All of the contigs and singleton reads were homologous to AdeV species C, which is commonly associated with RTI and gastroenteristis [66], sharing 100% amino acid identity for local alignments.

#### Human Rotavirus

Group A human rotaviruses (RVA) are contagious viruses responsible for acute gastroenteritis and severe watery diarrhea and found at high prevalence in infants, young children and immunocompromised individuals [67, 68]. Currently, RVA classification is based on phylogenetic relations of the outermost layer of the structural genes VP7 (G type) and VP4 (P type), referred to as G/P genotypes. Sequences similar to RVA were detected in the HFMD01 pool with a total number of 30 reads, assembling into 10 contigs (171 nt to 338 nt) and 2 singletons (172 nt and 178 nt). The fragments covered 4 of the structural genes including VP2, VP3, VP6, and VP7 and nonstructural genes NSP1 and NSP4. Phylogenetic analyses showed that the RVA-like strain were clustered within lineage 1 (G1-I1-C1-M1-A1-N1 genes constellations) with nucleotide sequence identity 98%-99% to the references in GenBank.

#### Human Picobirnavirus

Human picobirnavirus (HPBV) are non-enveloped, spherical viruses with two double-stranded RNA genomes. The virus has frequently been found in human feces as environmental contaminants as well as a pathogen in outbreaks of acute gastroenteritis and diarrhea [69]. However, clinical importance of the viruses remains unclear. The HFMD-lib03 pool contained 29 reads which could be assembled into 3 contigs (208 nt, 307 nt, and 531 nt) with 62%-77% amino acid identity to the RdRp gene of HPBV and the 2 singleton reads (121 nt and 163 nt) showed the best hit to the capsid protein genes of the different HPBV strains with 46%-67% amino acid identity. Study of enteric virus infections in infants showed that HPBV was one of the most common viruses frequently detected over a period of time [70]. In this study, the sequence we recovered was relatively distant from previously reported HPBV sequences, and its classification was unclear.

#### Human Respiratory Syncytial Virus

Human respiratory syncytial virus (RSV) belongs to the genus *Pneumovirus*, family *Paramyxoviridae*. RSV is one of the leading causes of lower RTI in infants and hospitalized children [41, 71] and associated with the subsequent development of childhood asthma [72]. Although there is no evidence indicating that RSV is an enteric pathogen, the HFMD-lib03 pool in this study contained sequences similar to RSV, which constituted 2.2% (187/8,482) of the virome. Compared to their closest database match, all of the sequences aligned with RSV species A and showed nucleotide sequence identity between 84% and 100%, while the amino acid sequence identities varied between 78% and 100% to other RSV type A

#### Human Polyomavirus

In HFMD virome, 2 contigs of 5 reads (195 nt and 257 nt) and 1 singlet (158 nt) were identified as BK polyomavirus (BKV) with approximately 99% nucleotide similarity to the previous sequences. BKV is a non-enveloped virus with a circle dsDNA genome that belongs to the *Papovaviridae* family. BKV has been detected in a wide range of tissue types and can persist without causing symptoms [73]. BKV can establish latent infection of the kidneys and causes neuropathy and nephropathy in immunosuppressed transplant patients [74, 75]. It has been suggested that the gastrointestinal tract is the latency site for BKV [76]. In addition, BKV can cause colonic ulceration in kidney transplant patient under immunosuppressive drug [77]. So far, route of transmission of BKV has not been clearly determined. However, accumulating evidence suggests that BKV can also be detected in the stool samples of hospitalized children and from gastroenteritis patients as well as healthy adults [78, 79].

#### Re-evaluation of enteroviruses among individual samples in HFMD-lib01, HFMD-lib02, and HFMD-lib03

Given the observation that picornavirus sequences generated high number of reads in all 3 pools examined by deep sequencing, we asked whether type-specific enteroviruses could be identified among the individual samples comprising each pool. Towards this, we tested each sample for the virus identified from the most frequent virus reads found in each pool using RT-PCR. For HFMD-lib01, we found one sample positive for CV-A16 after conventional sequencing and BLAST analysis, while the rest of the samples in this pool tested negative. In pool HFMD-lib02, which showed high number of reads for rhinovirus-C, we also found that only one sample tested positive for rhinovirus. Finally, we identified one sample positive for CV-A21 in HFMD-lib03 pool. Taken together, these results verified that the presence of enterovirus originated from a unique sample and contributed to the high number of reads observed in each pool.

## Discussion

Our study characterized the virome in fecal samples of pediatric HFMD patients during a 2012 widespread outbreak in Thailand. We used high-throughput next-generation sequencing to better understand the viruses present in HFMD patients who tested negative for enterovirus species A EV-71 and CV-A16/A6, predominant causes of HFMD and the main focus of many existing diagnostic assays [26, 27]. Analysis of the sequences either assembled into contigs or as singletons revealed a complexity of the virus population. Although previous examination of these samples did not initially detect enteroviruses using PCR-based assays, the present study utilizing deep sequencing enabled the detection of enterovirus sequences as well as other enteric virus at concentration as low as a few genome copies, providing a more systematic analysis of the prevalence of the viral genome.

The majority of viral reads identified here belonged to the family *Picornaviridae*, and were dominated by several members of the genus *Enterovirus*, namely HRV-C, CV-A21, and CV-A10. In addition to the commonly identified EV71 and CV-A16, other enteroviruses have been reported to cause HFMD outbreaks in different countries including CV-A10 and CV-A6 in Finland [5] and France [80] in 2010 and in China during 2008–2012 [81]. CV-A21 was least reported to cause HFMD with only 42 detections throughout the 36 years of surveillance in the United States [8]. However, a study in China cited a high incidence rate of CV-A21 infection in adults with RTI [82]. Another rarely detected virus found was EV68, originally isolated in the USA in 1962 from patients with RTI [83] and has been detected sporadically thereafter. EV68 is unusual among EV in that it shares phenotypic properties of both enterovirus and rhinovirus [84]. Reports suggested that this virus was associated with RTI including pneumonia and bronchiolitis with greater severity in infant and school-aged children. Between 2008 and 2010, an increasing number of EV68 clusters in cases of RTI have been reported worldwide [85–88]. Outbreaks have also been reported in the USA in 2014 when EV68 infection affected more than a thousand people, mostly young children and resulting in at least 12 deaths. The association of EV68 infection and diseases other than RTI remains uncertain. Studies have linked the virus with infection of the central nervous system including acute flaccid paralysis [8] and fatal meningomyeloencephalitis [89]. In addition, by using un-biased metagenomic analysis, our results not only expanded the number of identified types of enterovirus, both common and rare, but also revealed possible circulation of multiple enterovirus types during the outbreak.

Nearly completed genome of HRV species C was detected in the virome. Although the ability for HRV to replicate in the gastrointestinal tract is unknown, the detection of HRV in fecal samples has been reported [42, 43]. The amount of HRV RNA detected in feces could be as abundant as enteroviruses [90] and feces-derived HRV can retain infectivity in cell culture [91, 92]. No role for HRV has been postulated for HFMD and the virus is typically present in respiratory secretion and associated with asthma exacerbation [93]. Nucleic acids from HRV and other typical respiratory viruses such as RSV in feces may reflect inactivated particles in swallowed respiratory secretions or actual replication in the digestive tract possibly aided by adaptive mutations [90].

The second most abundant viral sequences identified belonged to astroviruses. The classic HAstV species consisting of eight serotypes has been associated with diarrhea particularly in immunodeficient patients [94]. Astroviruses detected here belonged to recently described species MLB1 and MLB2. MLB1 was initially sequenced from the fecal samples of a child with diarrhea [95] and serological survey revealed it to be a common childhood infection [96]. A study of children in India failed to associate MLB1 with diarrhea [97]. Meanwhile, MLB2 was also initially detected in fecal samples from Indian children with diarrhea [98] and in the plasma of a child with undifferentiated fever [97], suggesting replication beyond the digestive tract. Other astroviruses in human and animal tissues have also indicated likely neurological involvement [99–101], therefore a wide range of diseases may be associated with astrovirus infections.

Viral metagenomic studies of human fecal samples have been reported [102–105] and had suggest evidence of frequent co-infections with known enteric viruses from the family *Picornaviridae*, *Astroviridae*, and *Parvoviridae* [106, 107]. Our study found enterovirus, cardiovirus, astrovirus, rotavirus, norovirus, adenovirus, bocavirus, and picobirnavirus in stools from pediatric HFMD patients. Several of these viruses are known to cause gastrointestinal diseases. Although co-infections with different viruses and correlates of disease severity are ongoing, it is conceivable that viral co-infections may aggravate clinical manifestation of an otherwise mild virus infection, which could result in compromised gut function and upregulated immune response leading to increased risk of morbidity. For example, during an HFMD outbreak in Sarawak, Malaysia in 1997, a subgroup of adenovirus and enterovirus were isolated from three fatal cases [108]. It is therefore conceivable that co-infection could precipitate or aggravate HFMD symptoms. Future studies to examine viral profile in individual clinical samples are warranted to clarify the possible role of co-infection with disease severity.

Since a significant fraction of HFMD cases remains negative for the viral enterovirus pathogens and a direct or aggravating role for other viruses is conceivable, our study illustrates an overview of the virus community in stools from pediatric HFMD patients using unbiased sequencing approach. Our finding shows that such EV71, CV-A16/A6 PCR-negative children shed multiple types of picornaviruses and other enteric viruses in their feces including enterovirus, cardiovirus, astrovirus, rotavirus, norovirus, adenovirus, bocavirus, and picobirnavirus. Such overview information may help clinicians figure out the contribution of different types of picornaviruses as well as other pathogens to HFMD, with potential public health implications on the disease control.

The analysis of pooled samples and the deep sequencing method used presented limitations in the interpretation of the results. First, it was not possible to equate viral read numbers with the number of children infected. Second, comparison of the viral loads of viruses with different genome types (e.g. ssRNA, dsRNA, dsDNA) was not possible since the relative efficiency of converting their genomes into next-generation sequencing-compatible DNA may vary. Although stool samples used in this study were convenient samples collected for our previous study, inclusion of other clinical specimens such as throat swab, vesicular fluid, and skin lesions would be ideal. Stool samples may harbor unknown inhibitors of PCR and present nucleic acids of non-viral origin, thus complicating analysis. Nevertheless, our findings highlight the potential advantage of next generation sequencing to detect viruses from clinical specimens, which may be present below the limit of detection by conventional PCR assay. Assessing the role, if any, of these viruses in HFMD will require the study of larger populations, including epidemiologically matched healthy controls, and the analysis of individual, rather than pooled, clinical samples.

## Supporting Information

S1 Table General information of the pool sample collected from patients with hand, foot and mouth disease. (DOCX)

S2 Table Recombination analysis with P-value < 1E^-5^ of the reference set and the SAFV strain identified in the present study using RDP4 package. Results are shown for all recombination events with P <1E^-5^ by at least two analyses mode.
(DOCX)
