# Supplementary material for: The Fecal Virome of Children with Hand, Foot, and Mouth Disease that Tested PCR Negative for Pathogenic Enteroviruses
Source: PLoS One. 2015 Aug 19;10(8):e0135573. doi: 10.1371/journal.pone.0135573 (PMC4545796; doi:10.1371/journal.pone.0135573)
Supplement: S1 Table — (DOCX) [file pone.0135573.s001.docx]

**S1 Table. General information of the pool sample collected from patients with hand, foot and mouth disease.**

| **Library** | **Code** | **Patients (Sex/ Age)** | **Date** | **List of Detected Viruses** | |
| --- | --- | --- | --- | --- | --- |
|  |  |  |  | **Eukaryotic viruses** | **Picornaviruses** |
| HFMD-lib01 | A228 | Female 32 months | 21/6/2012 | Picornaviruses (1,244)  Bocaparvovirus (1,291)  Astrovirus (10)  Calicivirus (7)  Adenovirus (9)  Reovirus (30 | SAFV (788)  CV-A10 (433)  CV-A16 (17)  EV71 (3)  EV68 (3) |
|  | A230 | Male 8 months | 21/6/2012 |  |  |
|  | A231 | Female 12 months | 21/6/2012 |  |  |
|  | A232 | Male 18 months | 21/6/2012 |  |  |
|  | A243 | Female 48 months | 22/6/2012 |  |  |
|  | A297 | Male 32 months | 27/6/2012 |  |  |
|  | A298 | Male 30 months | 27/6/2012 |  |  |
|  | A303 | Male 48 months | 28/6/2012 |  |  |
|  | A398^a^ | Female 9 months | 4/7/2012 |  |  |
|  | A417 | Male 41 months | 5/7/2012 |  |  |
| HFMD-lib02 | A440 | Female 33 months | 10/7/2012 | Picornaviruses (1,784)  Calicivirus (11)  Adenovirus (3) | HRV-C (1,772) CV-A8 (6)  SAFV (4)  CV-A21 (2) |
|  | A445 | Male 13 months | 10/7/2012 |  |  |
|  | A511 | Male 48 months | 13/7/2012 |  |  |
|  | A512 | Female 33 months | 13/72012 |  |  |
|  | A513 | Male 11 months | 13/7/2012 |  |  |
|  | A529 | Female 20 months | 13/7/2012 |  |  |
|  | A530 | Female 18 months | 13/7/2012 |  |  |
|  | A532 | Female 55 months | 16/7/2012 |  |  |
|  | A537 | Female 24 months | 16/7/2012 |  |  |
|  | A539 | Female 60 months | 16/7/2012 |  |  |
| HFMD-lib03 | A545 | Female 18 months | 16/7/2012 | Picornaviruses (1,678)  Astrovirus (1,469)  Calicivirus (703)  Paramyxovirus (187)  Adenovirus (21)  Picobirnavirus (29)  Polyomavirus (6) | CV-A21 (1,334)  CV-B1 (125)  EV-B (200)  Echo3 (17)  EV-A (2) |
|  | A546 | Female 10 months | 16/7/2012 |  |  |
|  | A548 | Male 9 months | 16/7/2012 |  |  |
|  | A549 | Female 18 months | 16/7/2012 |  |  |
|  | A556 | Female 36 months | 17/7/2012 |  |  |
|  | A561 | Female 24 months | 17/7/2012 |  |  |
|  | A974 | Female 60 months | 24/10/2012 |  |  |
|  | A976 | Male 60 months | 26/10/2012 |  |  |
|  | A977 | Male 60 months | 26/10/2012 |  |  |

^a^ All clinical samples were collected from hospitals located in Bangkok, except sample A398 was collected from a hospital in Khon Kaen province.
