# Supplementary material for: The Fecal Virome of Children with Hand, Foot, and Mouth Disease that Tested PCR Negative for Pathogenic Enteroviruses
Source: PLoS One. 2015 Aug 19;10(8):e0135573. doi: 10.1371/journal.pone.0135573 (PMC4545796; doi:10.1371/journal.pone.0135573)
Supplement: S2 Table — Results are shown for all recombination events with P <1E-5 by at least two analyses mode. (DOCX) [file pone.0135573.s002.docx]

**S2 Table. Recombination analysis with P-value < 1E-5 of the reference set and the SAFV strain identified in the present study using RDP4 package. Results are shown for all recombination events with P <1E^-5^ by at least two analysis modes.**

| No | Event | Parental and Recombinant Strains | | | | | | Genomic Region | Breaking point | | P-Value | | | | | | |
| --- | --- | --- | --- | --- | --- | --- | --- | --- | --- | --- | --- | --- | --- | --- | --- | --- | --- |
|  |  | Major | Genotype | Minor | Genotype | Recombinant | Genotype |  | Start | End | RDP | GENECONV | BootScan | MaxChi | Chimerara | Siscan | 3Seq |
| 1 | 1 | AB747258 | SAFV-11 | JX163901 | SAFV-2 | GU595289 | SAFV-2 | VP4, 2A | 1412 | 3961 | 6.68e-16 |  | 3.01e-35 | 1.42e-27 | 3.10e-19 | 1.05e-52 | 4.07e-07 |
| 2 |  | AB747258 | SAFV-11 | JX163901 | SAFV-2 | NC_010810 | SAFV-2 | VP4, 2A | 1429 | 3968 | 6.68e-16 |  | 3.01e-35 | 1.42e-27 | 3.10e-19 | 1.05e-52 | 4.07e-07 |
| 3 |  | AB747258 | SAFV-11 | JX163901 | SAFV-2 | EU376394 | SAFV-2 | VP4, 2A | 1429 | 3968 | 6.68e-16 |  | 3.01e-35 | 1.42e-27 | 3.10e-19 | 1.05e-52 | 4.07e-07 |
| 4 |  | AB747258 | SAFV-11 | JX163901 | SAFV-2 | JF813004 | SAFV-2 | VP4, 2B | 1429 | 3968 | 6.68e-16 |  | 3.01e-35 | 1.42e-27 | 3.10e-19 | 1.05e-52 | 4.07e-07 |
| 5 |  | AB747258 | SAFV-11 | JX163901 | SAFV-2 | AM922293 | SAFV-2 | 5UTR, 2A | 1437 | 3970 | 6.68e-16 |  | 3.01e-35 | 1.42e-27 | 3.10e-19 | 1.05e-52 | 4.07e-07 |
| 6 | 2 | AB747258 | SAFV-11 | FJ463615 | SAFV-5 | FJ463616 | SAFV-5 | VP4, 2A | 1512 | 4254 |  |  | 1.05e-30 | 2.79e-26 | 4.16e-23 | 5.58e-65 | 6.64e-26 |
| 7 |  | AB747258 | SAFV-11 | FJ463615 | SAFV-5 | FJ463615 | SAFV-5 | VP4, 2A | 1512 | 4257 |  |  | 1.05e-30 | 2.79e-25 | 4.16e-23 | 5.58e-65 | 6.64e-26 |
| 8 | 3 | EF165067 | SAFV-1 | HM181996 | SAFV-3 | HM181999 | SAFV-3 | VP4, 2A | 1531 | 3987 | 8.45e-12 |  | 4.08e-17 | 5.33e-12 | 8.48e-15 | 2.71e-42 | 2.42e-12 |
| 9 |  | EF165067 | SAFV-1 | HM181996 | SAFV-3 | HM181997 | SAFV-3 | VP4, 2A | 1531 | 3987 | 8.45e-12 |  | 4.08e-17 | 5.33e-12 | 8.48e-15 | 2.71e-42 | 2.42e-12 |
| 10 |  | EF165067 | SAFV-1 | HM181996 | SAFV-3 | HM181998 | SAFV-3 | VP4, 2A | 1432 | 4226 | 8.45e-12 |  | 4.08e-17 | 5.33e-12 | 8.48e-15 | 2.71e-42 | 2.42e-12 |
| 11 |  | EF165067 | SAFV-1 | HM181996 | SAFV-3 | EU681178 | SAFV-3 | VP4, VP1 | 1527 | 3438 | 8.45e-12 |  | 4.08e-17 | 5.33e-12 | 8.48e-15 | 2.71e-02 | 2.42e-12 |
| 12 |  | EF165067 | SAFV-1 | HM181996 | SAFV-3 | EU681179 | SAFV-3 | VP4, VP1 | 1542 | 3453 | 8.45e-12 |  | 4.08e-17 | 5.33e-12 | 8.48e-15 | 2.71e-42 | 2.42e-12 |
| 13 | 4 | EU681176 | SAFV-2 | HM181999 | SAFV-3 | EU681178 | SAFV-3 | 5UTR, 2A | 77 | 4119 | 8.33e-24 | 1.29e-22 | 4.13e-45 | 7.08e-31 | 1.38e-19 | 1.06e-78 |  |
| 14 | 5 | EU681176 | SAFV-2 | EU681178 | SAFV-3 | HFMD-SAFV | SAFV-3 | VP2, 2A | 801 | 4137 | 2.12e-14 |  | 3.58e-45 | 5.89e-26 | 2.55e-24 | 5.54e-64 | 3.59e-24 |
| 15 |  | EU681176 | SAFV-2 | EU681178 | SAFV-3 | HQ162476 | SAFV-3 | VP4, 2A | 1429 | 4042 | 2.12e-14 |  | 2.58e-45 | 5.89e-26 | 2.55e-24 | 5.54e-64 | 3.59e-24 |
| 16 |  | EU681176 | SAFV-2 | EU681178 | SAFV-3 | FM207487 | SAFV-3 | L, 2A | 1303 | 4205 | 2.12e-14 |  | 3.58e-45 | 5.89e-26 | 2.55e-24 | 5.54e-64 | 3.59e-24 |
| 17 |  | EU681176 | SAFV-2 | EU681178 | SAFV-3 | GU943513 | SAFV-3 | VP4, 2A | 1465 | 4205 | 2.12e-14 |  | 3.58e-45 | 5.89e-26 | 2.55e-24 | 5.54e-64 | 3.59e-24 |
| 18 |  | EU681176 | SAFV-2 | EU681178 | SAFV-3 | HQ902242 | SAFV-3 | VP4, 2A | 1429 | 4137 | 2.12e-14 |  | 3.58e-45 | 5.89e-26 | 2.55e-24 | 5.54e-64 | 3.59e-24 |
| 19 |  | EU681176 | SAFV-2 | EU681178 | SAFV-3 | GU943514 | SAFV-3 | L, 2A | 1408 | 4137 | 2.12e-14 |  | 3.58e-45 | 5.89e-26 | 2.55e-24 | 5.54e-64 | 3.59e-24 |
| 20 | 6 | EU681177 | SAFV-2 | GU595289 | SAFV-2 | AM922293 | SAFV-2 | 5UTR | 1145 | 1428 | 6.19e-17 |  | 2.83e-13 |  |  | 9.87e-09 | 9.79e-19 |
| 21 | 7 | FJ463615 | SAFV-5 | EU681177 | SAFV-2 | GU943518 | SAFV-2 | VP4, 2A | 1555 | 3951 |  |  | 5.97e-07 | 1.63e-12 | 3.90e-07 | 9.10e-24 |  |
| 22 | 8 | FM207487 | SAFV-3 | EU681177 | SAFV-2 | JN652231 | SAFV-2 | VP4, 2A | 1279 | 3642 | 5.70e-18 |  | 1.13e-37 | 2.93e-26 | 8.46e-21 | 2.21e-55 | 8.88e-28 |
| 23 | 9 | FM207487 | SAFV-3 | HM181999 | SAFV-3 | GU943513 | SAFV-3 | 5UTR | 13 | 525 |  | 2.08E-12 | 8.07e-23 | 3.10e-09 | 2.97e-10 | 1.15e-11 | 2.16e-14 |
| 24 | 10 | GU595289 | SAFV-2 | EU681177 | SAFV-2 | GU595289 | SAFV-2 | 5UTR, L, VP4 | 286 | 1392 | 6.19e-17 |  | 2.83e-13 |  |  | 9.87e-09 | 9.79e-19 |
| 25 |  | GU595289 | SAFV-2 | EU681177 | SAFV-2 | JF813004 | SAFV-2 | 5UTR, L | 403 | 1355 | 6.19e-17 |  | 2.83e-13 |  |  | 9.87e-09 | 9.79e-19 |
| 26 |  | GU595289 | SAFV-2 | EU681177 | SAFV-2 | EU376394 | SAFV-2 | 5UTR, L, VP4 | 286 | 1392 | 6.19e-17 |  | 2.83e-13 |  |  | 9.87e-09 | 9.79e-19 |
| 27 | 11 | GU595289 | SAFV-2 | HM181996 | SAFV-3 | NC_010810 | SAFV-2 | 5UTR | 128 | 600 |  |  | 8.14e-07 |  |  | 9.53e-06 |  |
| 28 |  | GU595289 | SAFV-2 | HM181996 | SAFV-3 | EU376394 | SAFV-2 | 5UTR | 128 | 600 |  |  | 8.14e-07 |  |  | 9.53e-06 |  |
| 29 |  | GU595289 | SAFV-2 | HM181996 | SAFV-3 | JF813004 | SAFV-2 | 5UTR | 113 | 237 |  |  | 8.14e-07 |  |  | 9.53e-06 |  |
| No | Event | Parental and Recombinant Strains | | | | | | Genomic Region | Breaking point | | P-Value | | | | | | |
|  |  | Major | Genotype | Minor | Genotype | Recombinant | Genotype |  | Start | End | RDP | GENECONV | BootScan | MaxChi | Chimerara | Siscan | 3Seq |
| 30 | 12 | HM181997 | SAFV-3 | EU681177 | SAFV-2 | FN999911 | SAFV-2 | VP4, 2A | 1579 | 4026 | 5.70e-18 |  | 1.13e-37 | 2.93e-26 | 8.46e-21 | 2.21e-55 | 8.88e-28 |
| 31 |  | HM181997 | SAFV-3 | EU681177 | SAFV-2 | JN652232 | SAFV-2 | VP4, 2A | 1573 | 4008 | 5.70e-18 |  | 1.13e-37 | 2.93e-26 | 8.46e-21 | 2.21e-55 | 8.88e-28 |
| 32 |  | HM181997 | SAFV-3 | EU681177 | SAFV-2 | JN652233 | SAFV-2 | VP4, 2A | 1573 | 4008 | 5.70e-18 |  | 1.13e-37 | 2.93e-26 | 8.46e-21 | 2.21e-55 | 8.88e-28 |
| 33 |  | HM181997 | SAFV-3 | EU681177 | SAFV-2 | EU681176 | SAFV-2 | VP4, 2A | 1579 | 4026 | 5.70e-18 |  | 1.13e-37 | 2.93e-26 | 8.46e-21 | 2.21e-55 | 8.88e-28 |
| 34 |  | HM181997 | SAFV-3 | EU681177 | SAFV-2 | JX163901 | SAFV-2 | VP2, 2B | 1391 | 4321 | 5.70e-18 |  | 1.13e-37 | 2.93e-26 | 8.46e-21 | 2.21e-55 | 8.88e-28 |
| 35 | 13 | HQ902242 | SAFV-3 | AB747258 | SAFV-11 | NC_009448 | SAFV-1 | 5UTR, VP4 | 132 | 1431 |  |  | 1.76e-12 |  |  | 4.77e-21 |  |
| 36 |  | HQ902242 | SAFV-3 | AB747258 | SAFV-11 | EF165067 | SAFV-1 | 5UTR, VP4 | 132 | 1431 |  |  | 1.76e-12 |  |  | 4.77e-21 |  |
| 37 | 14 | JX163901 | SAFV-2 | FJ463616 | SAFV-5 | FJ463617 | SAFV-6 | 2C, 3C | 4939 | 6660 |  |  | 9.38e-08 | 2.81e-09 | 7.82e-06 | 1.70e-21 |  |
| 38 | 15 | NC_009448 | SAFV-1 | AB747250 | SAFV-3 | AB747248 | SAFV-1 | 2B, 3D | 4294 | 7024 | 3.31e-30 |  | 2.25e-26 | 2.25e-12 | 2.60e-07 | 7.17e-14 | 1.21e-34 |
| 39 | 16 | NC_009448 | SAFV-1 | EU681179 | SAFV-3 | HM181996 | SAFV-3 | VP4, 2A | 1574 | 4006 |  |  | 2.78e-06 | 9.53e-09 | 8.25e-10 | 5.08e-11 | 1.23e-08 |
| 40 | 17 | AB747257 | SAFV-10 | JX163901 | SAFV-2 | AB747249 | SAFV-2 | VP4, 2A | 1460 | 4064 |  |  | 1.47e-09 |  | 2.51e-08 | 5.24e-38 |  |
| 41 | 18 | AB747256 | SAFV-9 | AB747251 | SAFV-4 | AB747257 | SAFV-10 | 3B, RdRp | 5839 | 7012 | 1.16e-12 |  | 8.88e-16 |  |  | 6.23e-12 |  |
| 42 | 19 | GU943518 | SAFV-2 | AB747249 | SAFV-2 | AB747256 | SAFV-6 | 2B,RdRp | 4363 | 7456 |  |  | 2.78e-06 | 9.53e-09 | 8.25e-10 | 5.08e-11 | 1.23e-08 |
| 43 | 20 | EU681179 | SAFV-3 | AB747251 | SAFV-4 | AB747250 | SAFV-3 | 2A, 3D | 4124 | 6922 | 1.45e-15 |  | 5.47e-18 | 1.81e-16 | 1.60e-16 | 4.40e-11 | 1.95e-13 |
| 44 | 21 | GU943518 | SAFV-2 | AB747249 | SAFV-2 | AB747258 | SAFV-11 | 2C, 3D | 4942 | 6801 | 8.46e-11 |  | 3.53e-06 | 5.59e-08 |  | 8.47e-06 | 2.63e-09 |
| 45 | 22 | FJ463616 | SAFV-5 | AB747258 | SAFV-11 | AB747252 | SAFV-5 | 2C, 3D | 5209 | 6730 | 3.82e-38 |  | 4.84e-39 | 2.76e-12 | 8.07e-07 | 1.89e-10 | 6.24e-15 |
